# Immunogenicity and Reactogenicity of Coadministration of COVID-19 and Influenza Vaccines

**DOI:** 10.1001/jamanetworkopen.2023.32813

**Published:** 2023-09-08

**Authors:** Tal Gonen, Noam Barda, Keren Asraf, Gili Joseph, Yael Weiss-Ottolenghi, Ram Doolman, Yitshak Kreiss, Yaniv Lustig, Gili Regev-Yochay

**Affiliations:** 1 Sheba Pandemic Research Institute, Sheba Medical Center, Tel Hashomer, Ramat Gan, Israel; 2 The Infection Prevention & Control Unit, Sheba Medical Center, Tel Hashomer, Ramat Gan, Israel; 3 ARC Innovation Center, Sheba Medical Center, Tel Hashomer, Ramat Gan, Israel; 4 Software and Information Systems Engineering, Ben-Gurion University of the Negev, Be’er Sheva, Israel; 5 Epidemiology, Biostatistics and Community Health Services, Ben-Gurion University of the Negev, Be’er Sheva, Israel; 6 The Dworman Automated-Mega Laboratory, Sheba Medical Center, Tel-Hashomer, Ramat-Gan, Israel; 7 Sackler School of Medicine, Tel-Aviv University, Tel Aviv, Israel; 8 General Management, Sheba Medical Center, Tel Hashomer, Ramat Gan, Israel; 9 Central Virology Laboratory, Public Health Services, Ministry of Health, Tel-Hashomer, Ramat Gan, Israel

## Abstract

**Importance:**

COVID-19 and seasonal influenza vaccines were previously given separately, although their coadministration is warranted for vaccination adherence. Limited data on their coadministration have been published.

**Objective:**

To compare the reactogenicity and immunogenicity of COVID-19 and influenza vaccinations administered together with those of COVID-19 vaccination alone.

**Design, Setting, and Participants:**

This prospective cohort study included health care workers at a large tertiary medical center in Israel who received the Influvac Tetra (Abbott) influenza vaccine (2022/2023), the Omicron BA.4/BA.5–adapted bivalent (Pfizer/BioNTech) vaccine, or both. Vaccination began in September 2022, and data were collected until January 2023. Vaccines were offered to all employees and were coadministered or given separately. Adverse reaction questionnaires were sent, and serologic samples were also collected.

**Exposures:**

Receiving COVID-19 vaccine, influenza vaccine, or both.

**Main Outcomes and Measures:**

The main outcomes for the reactogenicity analysis were symptoms following vaccine receipt, assessed by a digital questionnaire: any local symptoms; fever; weakness or fatigue; any systemic symptoms; and their duration. The immunogenicity analysis’ outcome was postvaccination anti-spike IgG titer.

**Results:**

This study included 2 cohorts for 2 separate analyses. The reactogenicity analysis included 588 participants (of 649 questionnaire responders): 85 in the COVID-19 vaccine–alone group (median [IQR] age, 71 [58-74] years; 56 [66%] female); 357 in the influenza vaccine–alone group (median [IQR] age, 55 [40-65] years; 282 [79%] female); and 146 in the coadministration group (median [IQR] age, 61 [50-71] years; 81 [55%] female). The immunogenicity analysis included 151 participants: 74 participants in the COVID-19 vaccine group (median [IQR] age, 67 [56-73] years; 45 [61%] female) and 77 participants in the coadministration group (median [IQR] age, 60 [49-73] years; 42 [55%] female). Compared with COVID-19 vaccination alone, the risk of systemic symptoms was similar in the coadministration group (odds ratio, 0.82; 95% CI, 0.43-1.56). Geometric mean titers in the coadministration group were estimated to be 0.84 (95% CI, 0.69-1.04) times lower than in the COVID-19 vaccine–alone group.

**Conclusions and Relevance:**

In this cohort study of health care workers who received a COVID-19 vaccine, an influenza vaccine, or both, coadministration was not associated with substantially inferior immune response or to more frequent adverse events compared with COVID-19 vaccine administration alone, supporting the coadministration of these vaccines.

## Key Points

**Question:**

Is the coadministration of a COVID-19 vaccine with a seasonal influenza vaccine safe and efficacious?

**Findings:**

This prospective cohort study included health care workers vaccinated against COVID-19 and/or influenza. Compared with COVID-19 vaccination alone, the risk of systemic symptoms was lower and statistically insignificant in the coadministration group. Lower, statistically insignificant anti-spike IgG titers were found in the coadministration group.

**Meaning:**

In this study, both reactogenicity and immunogenicity were mostly unchanged with coadministration of the COVID-19 and season influenza vaccines compared with the administration of COVID-19 vaccination alone.

## Teaser

This cohort study compares the reactogenicity and immunogenicity of COVID-19 and influenza vaccinations administered together with those of COVID-19 vaccination alone.

## Introduction

SARS-CoV-2 vaccine boosters have been shown to reduce morbidity and mortality, as the effectiveness of the original vaccine regimen wanes over time.^1^ Boosters are of importance in the setting of health care workers (HCWs) as they come in contact with populations that are susceptible to developing severe COVID-19 symptoms and as they may also be caring for patients who are themselves infected with SARS-CoV-2. Early in the pandemic, certain public health organizations recommended that COVID-19 and seasonal influenza vaccinations (SIVs) be administered separately.^2^ However, during the 2022 to 2023 influenza season the US Centers for Disease Control and Prevention and other organizations recommended coadministration of these vaccines,^3^ aiming to reduce the burden on the health care system and increase adherence to vaccination. Several studies on the coadministration of COVID-19 vaccines and SIVs have been published,^4,5,6,7,8,9^ suggesting that adverse events occur at a similar rate when COVID-19 vaccines are administered alone and together with SIVs. Investigations of the immune response against influenza strains showed that it was mostly preserved between intervention and control groups.^4,5,6,8^ Some of these studies have found that the humoral response to SARS-CoV-2 was slightly reduced or unchanged when COVID-19 vaccination was administered with an SIV.^4,5^ However, these reports assessed the primary COVID-19 vaccination regimen and not the booster doses. Moreover, data on the coadministration of the Omicron BA.4/BA.5–adapted bivalent booster (Pfizer-BioNTech) vaccine with SIV have not been published, to our knowledge. The aim of this study is to compare the reactogenicity and immunogenicity of the COVID-19 Omicron BA.4/BA.5–adapted bivalent vaccine administered together with SIV with the reactogenicity and immunogenicity of this type of COVID-19 vaccination given alone in the context of a HCW population that had almost entirely previously received 1 or 2 COVID-19 vaccine booster doses.

## Methods

### Study Setting

This was a prospective cohort study which enrolled HCWs at a large tertiary medical center in Israel, the Sheba Medical Center (SMC). The SMC HCW cohort, from which participants in this study were enrolled, followed up HCWs through frequent serological tests beginning in December 2020, when a COVID-19 vaccination campaign began at SMC, as was previously described in detail.^10^ All HCWs who agreed to participate signed a written informed consent form in Hebrew. This cohort study was approved by the institutional review board at SMC. The study was funded by internal funding of The Sheba Pandemic Preparedness Research Institute and not sponsored or funded by any commercial entity. This study was performed following Strengthening the Reporting of Observational Studies in Epidemiology (STROBE) reporting guidelines.

**Figure 1.**
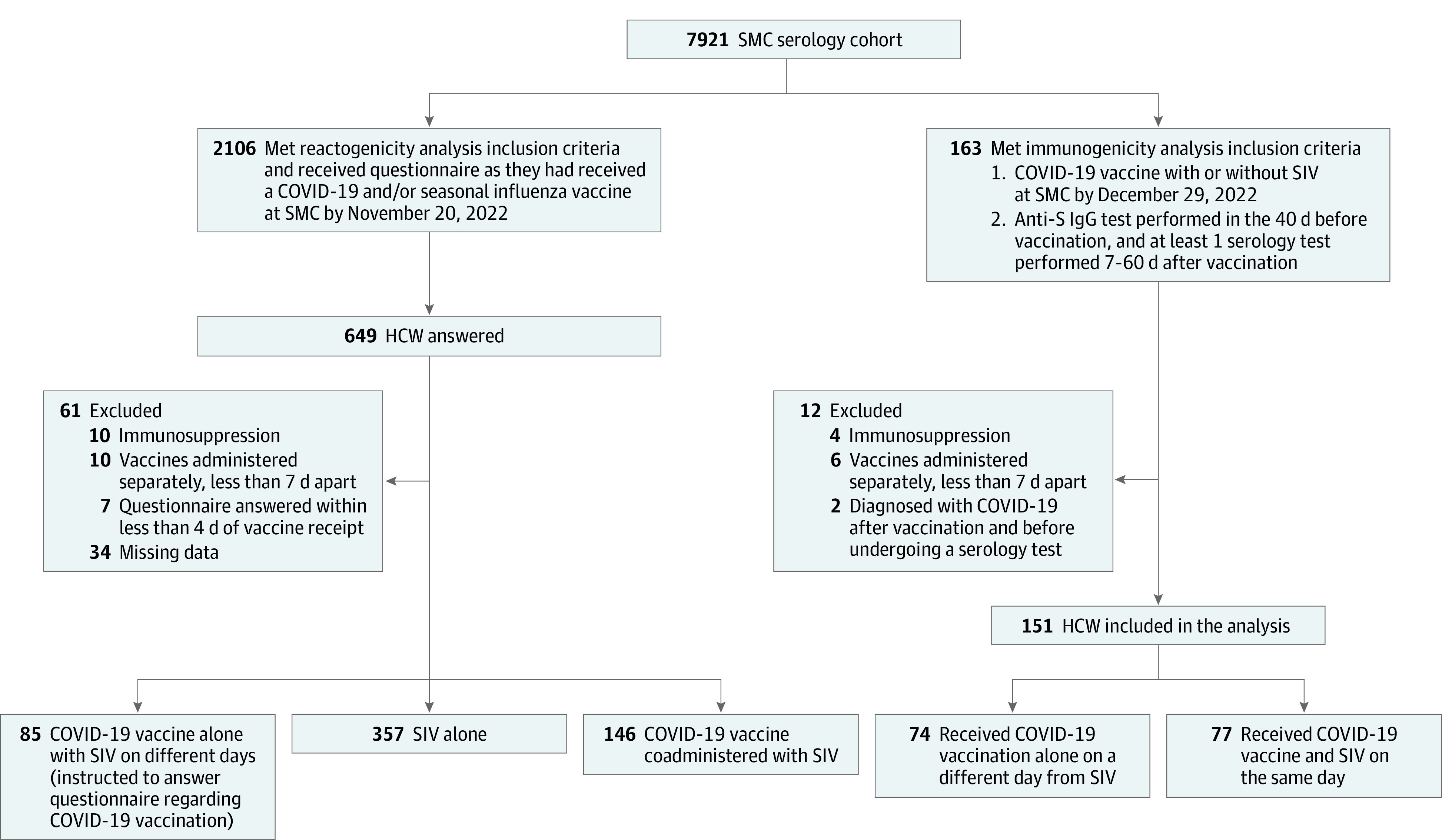
Study Population Flowchart Reactogenicity and immunogenicity analyses were carried out based on different inclusion and exclusion criteria. The cohorts of both analyses overlapped, with 88 health care workers (HCWs) included in both analyses. SIV indicates seasonal influenza vaccine; SMC, Sheba Medical Center.

In the fall of 2022, all HCWs at SMC were encouraged to vaccinate for COVID-19 with the Omicron BA.4/BA.5–adapted bivalent vaccine (henceforth COVID-19 vaccine) and for influenza with the Influvac Tetra SIV (Abbott) (2022/2023, henceforth SIV). These vaccines were offered as 2 shots administered together on a single day (injected into opposite arms), although vaccinees could opt to be administered only 1 of the vaccines or both, but on separate days. COVID-19 vaccination was administered in SMC starting September 28, 2022, while SIV was administered in SMC starting October 12, 2022. The reactogenicity analysis part of this trial enrolled HCWs who were vaccinated by November 29, 2022, and the immunogenicity part of this trial enrolled HCWs who were vaccinated by December 29, 2022. Figure 1 illustrates the study’s cohorts and their follow-up.

### Study Design

Reactogenicity was assessed by an electronic questionnaire sent up to 62 days after vaccination, addressing local and systemic symptoms (eTable 1 in Supplement 1). Inclusion criteria for this analysis were having received SIV and/or COVID-19 vaccines during the study period and participation in the SMC serology cohort. Exclusion criteria were immunosuppression (eMethods 1 in Supplement 1), having received both COVID-19 and SIV on separate days but within fewer than 7 days of each another, answering the electronic questionnaire less than 5 days after having received vaccination, and incomplete or incoherent responses to the questionnaire (such as reporting symptoms of COVID-19 vaccination while only receiving SIV according to SMC records). The exposures in this analysis were the vaccines received (COVID-19 vaccine alone, SIV alone, or both vaccines administered together). Those who received both vaccines but on separate days were instructed to address only their post–COVID-19 vaccine reactions when answering the questionnaire and were therefore included in the COVID-19 vaccine–alone group. The following outcomes were assessed and odds ratios (ORs) were calculated: any local symptoms (binary; defined as pain, swelling, redness, pruritus, or any other local reaction at the injection site); fever (binary; defined as fever ≥37.5 °C at any point); significant weakness or fatigue (binary); any systemic symptoms (binary; defined as fever, headache, myalgia, significant fatigue or weakness, lymphadenopathy, or any other systemic symptom); and duration of headache, myalgia, or significant weakness or fatigue (ordinal; no occurrence, <24 hours, 24-48 hours, 48-72 hours, 72-96 hours, or >96 hours).

Immunogenicity was assessed by postvaccination anti-spike IgG titers. Inclusion criteria for this analysis were having received COVID-19 vaccine with or without SIV during the study’s period, participation in the SMC serology cohort, and having undergone serology tests before (up to 40 days) and after (6-70 days) vaccination with the COVID-19 vaccine. Exclusion criteria for this analysis were immunosuppression (eMethods 2 in Supplement 1), a documented diagnosis of COVID-19 during the period between COVID-19 vaccine receipt and postvaccination serological test, and having received both COVID-19 and SIV on separate days but within less than 7 days of each other. The exposures for which immunogenicity was assessed were coadministration of both COVID-19 vaccine and SIV and vaccination with COVID-19 alone (HCWs who received both vaccines separately, at least 7 days apart, were classified for the purpose of this analysis as having received COVID-19 vaccination alone). The outcome in this analysis was postvaccination anti-spike IgG titers, measured using SARS-CoV-2 IgG II Quant (Abbott).^11^

### Statistical Analysis

In the reactogenicity analysis, the incidence of each outcome was estimated as the empirical proportion. Confidence intervals were estimated using the exact binomial test for binary outcomes and the Sison-Glaz method^12^ for ordinal outcomes. To estimate the ORs for each outcome between COVID-19 vaccination alone (the baseline) vs influenza vaccination alone and vs coadministration of both vaccines, we used multivariable regression. Logistic regression was used for binary outcomes, while an ordered logit model was used for the ordinal outcome. The model was adjusted for age, sex, and number of comorbidities. Missing data in number of comorbidities were multiply imputed 5 times, with the estimates from each complete data set pooled using Rubin rules.

For the immunogenicity analysis, postvaccination geometric mean titers (GMTs) were plotted as a function of time, with a restricted cubic spline superimposed as a smoother. The geometric mean ratio (GMR) between the different study groups was estimated using a multivariable linear regression adjusted for age, sex, prevaccination IgG, and the number of days between vaccination and the postvaccination IgG measurement (categorized as 7-13, 14-20, 21-27, 28-34, 35-41, and 42-60 days). IgG levels were log-transformed for the analysis. Repeated measurements were addressed by inclusion of a random intercept per individual.

Statistical significance was defined as α = 0.05. The statistical software used for analysis was R version 4.1.2 (R Project for Statistical Computing).

## Results

This study included 2 overlapping cohorts for 2 separate analyses: a reactogenicity analysis and an immunogenicity analysis (Figure 1). The reactogenicity analysis included 588 participants: 85 in the COVID-19 vaccine–alone group (median [IQR] age, 71 [58-74] years; 56 [66%] female); 357 in the SIV-alone group (median [IQR] age, 55 [40-65] years;282 [79%] female); and 146 in the coadministration group (median [IQR] age, 61 [50-71] years; 81 [55%] female). The immunogenicity analysis included 151 participants: 74 participants in the COVID-19 vaccine–alone group (median [IQR] age, 67 [56-73] years; 45 [61%] female) and 77 participants in the coadministration group (median [IQR] age, 60 [49-73] years; 42 [55%] female). Eighty-eight of those HCW who were included in the immunogenicity analysis were also included in the reactogenicity analysis. See Table 1 and eTable 2 in Supplement 1 for further details on population characteristics.

**Table 1.** Baseline Characteristics of Study Populations

Characteristic	Participants, No. (%)		
	COVID-19 vaccine alone	SIV alone	COVID-19 vaccine administered with SIV
**Reactogenicity analysis**			
Participants, No.	85	357	146
Sex			
Female	56 (66)	282 (79)	81 (55)
Male	29 (34)	75 (21)	65 (45)
Age, median (IQR), y	71 (58-74)	55 (40-65)	61 (50-71)
No. of COVID-19 vaccinations previously received			
1	2 (2)	8 (2)	3 (2)
2	0	14 (4)	3 (2)
3	14 (16)	184 (52)	40 (27)
4	69 (81)	145 (41)	100 (69)
Missing vaccination history data	0	6 (2)	0
No. of comorbidities			
0	35 (46)	171 (57)	60 (49)
1	17 (22)	75 (25)	31 (25)
≥2	24 (32)	53 (18)	32 (26)
Missing, No.	9	58	23
Days between vaccine receipt and response to questionnaire, mean (IQR)	24.7 (19.5)	29.3 (14.0)	30.2 (12.0)
Responded to questionnaire withing 30 d of vaccine receipt	55 (65)	169 (47)	67 (46)
**Immunogenicity analysis**			
Participants, No.	75	NA	77
Sex			
Female	45 (61)	NA	42 (55)
Male	29 (39)	NA	35 (45)
Age, median (IQR), y	67 (56-73)	NA	60 (49-73)
No. of COVID-19 vaccinations previously received		NA	
1	2 (3)	NA	0
2	4 (5)	NA	0
3	10 (14)	NA	19 (25)
4	58 (78)	NA	58 (75)
No. of comorbidities		NA	
0	23 (37)	NA	37 (51)
1	17 (27)	NA	17 (24)
≥2	23 (37)	NA	18 (25)
Missing comorbidity data, No.	11	NA	5

Abbreviations: NA, not applicable; SIV, seasonal influenza vaccine.

The reactogenicity questionnaire was sent to 2106 HCW who were part of the SMC cohort and had been vaccinated for COVID-19, influenza, or both and was answered by 649 (30.8% response rate) (eTable 3 in Supplement 1), of whom 588 met the eligibility criteria and were included in the analysis. Overall, 291 study participants (49%) responded to the questionnaire within a month of vaccine receipt, and only 9 HCWs (1.5%) included in this analysis responded to it 45 to 49 days after vaccine receipt. Mean time from vaccine receipt to questionnaire response was 28.9 (range, 5-57; median, 31) days for all analysis groups.

**Figure 2.**
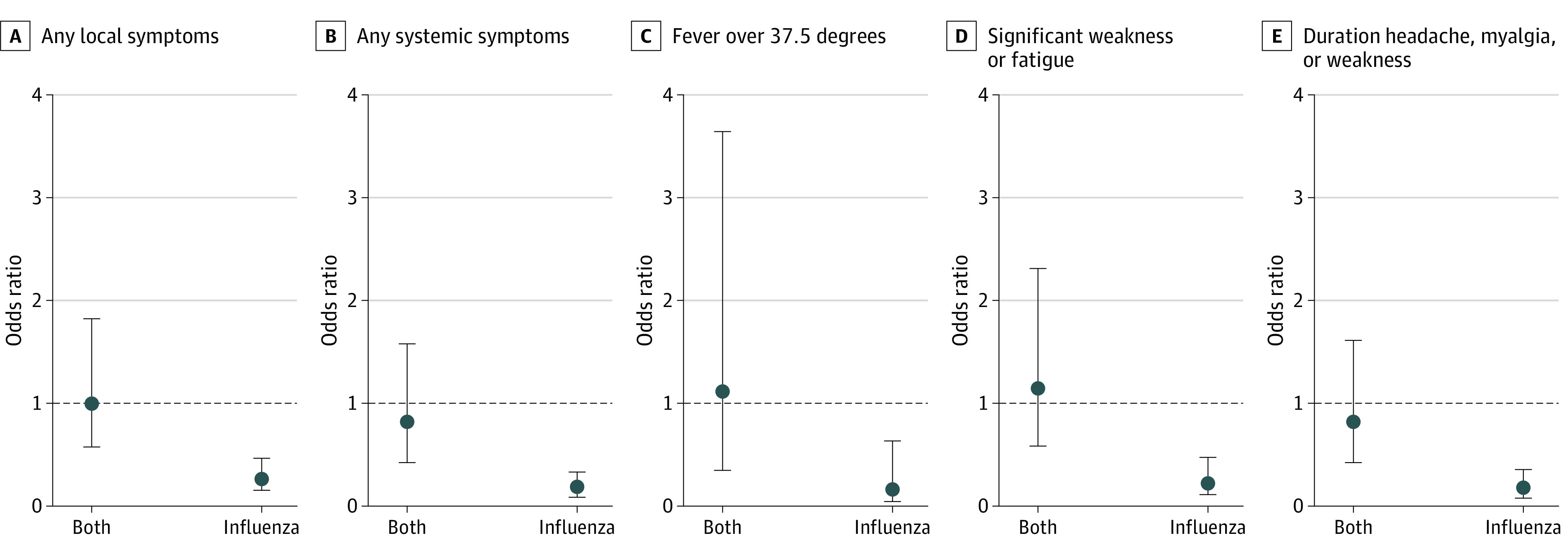
Estimated Odds of Adverse Events in the Coadministration Group (COVID-19 Vaccine and Seasonal Influenza Vaccine [SIV]) and SIV Group Compared With the COVID-19 Vaccine–Alone Group Logistic regression was used for binary outcomes, while an ordered logit model was used for the ordinal outcome. The model was adjusted for age, sex, and number of comorbidities. Missing data in number of comorbidities were multiply imputed 5 times, with the estimates from each complete data set pooled using Rubin rules.

The incidence of systemic reactions in the COVID-19 vaccination–alone group was 27.4% (95% CI, 18.2%-38.2%), 12.7% (95% CI: 9.5%-16.7%) in the SIV vaccination–alone, and 27.6% (95% CI: 20.5%-35.6%) in the coadministration group. Incidence rates of other adverse reactions are presented in Table 2. When comparing with the COVID-19 vaccination–alone group, risk of systemic symptoms was lower in the SIV group (OR, 0.17; 95% CI, 0.09-0.33), but similar in the coadministration group (OR, 0.82; 95% CI, 0.43-1.56) (Figure 2 and eTable 4 in Supplement 1).

**Table 2.** Incidence Proportion of Adverse Events^a^

Symptoms experienced	COVID-19 vaccine alone (n = 85)		SIV vaccine alone (n = 357)		COVID-19 vaccine coadministered with SIV (n = 146)	
	No.	Incidence (95 CI), %	No.	Incidence (95 CI), %	No.	Incidence (95 CI), %
Any local symptoms	42	49.4 (38.4-60.5)	123	34.5 (29.5-39.6)	76	52.1 (43.6-60.4)
Any systemic symptoms	23	27.4 (18.2-38.2)	45	12.7 (9.5-16.7)	40	27.6 (20.5-35.6)
Fever >37.5 °C	5	5.8 (2-13.3)	5	1.4 (0.5-3.3)	9	6.2 (2.9-11.5)
Significant weakness or fatigue	16	19 (11.3-29.1)	33	9.4 (6.6-12.9)	35	24.1 (17.4-31.9)
Duration of headache, myalgia, weakness, or fatigue						
No headache, myalgia, significant weakness or fatigue	61	74.4 (65.9-83.3)	308	89.5 (86.9-92.7)	105	72.9 (66.7-80.1)
<24 h	6	7.3 (0-16.3)	9	2.6 (0-5.8)	9	6.2 (0-13.5)
24-48 h	12	14.6 (6.1-23.6)	15	4.1 (1.5-7.2)	16	11.1 (4.9-18.3)
48-72 h	1	1.2 (0-10.2)	8	2.3 (0-5.5)	7	4.9 (0-12.1)
72-96 h	1	1.2 (0-10.2)	1	0.3 (0-3.4)	3	2.1 (0-9.3)
>96	1	1.2 (0-10.2)	4	1.2 (0-4.3)	4	2.8 (0-10.0)
Unknown	3	NA	13	NA	2	NA

Abbreviations: NA, not applicable; SIV, seasonal influenza vaccination.

^a^ The incidence of each outcome was estimated as the empirical proportion. Confidence intervals were estimated using the exact binomial test (for binary outcomes: any local symptoms, any systemic symptoms, fever >37.5 °C, significant weakness or fatigue) and the Sison-Glaz method (for ordinal outcomes: duration of headache, myalgia, weakness, or fatigue).

**Figure 3.**
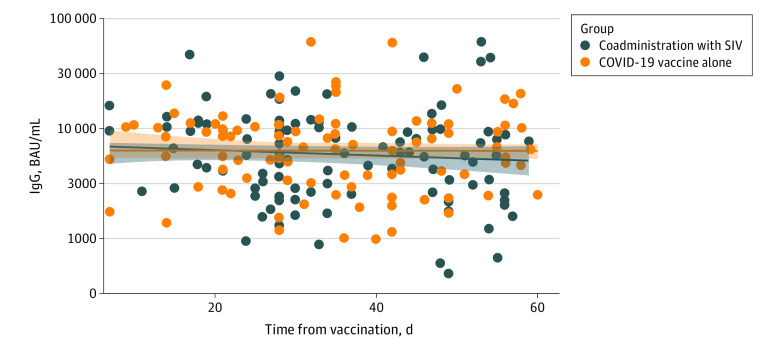
Postvaccination Anti-Spike IgG Geometric Mean Titers Plotted as a Function of Time Elapsed From Vaccination, COVID-19 Vaccine–Alone Compared With the Coadministration of COVID-19 With Seasonal Influenza Vaccine (SIV) The y-axis is log-transformed. A restricted cubic spline is superimposed as a smoother, with a 95% confidence band.

Altogether, 151 HCW were included in the immunogenicity analysis, contributing 214 postvaccination serological tests. Postvaccination GMTs in both exposure groups are plotted in Figure 3. GMTs in the coadministration group were estimated to be 0.84 (95% CI, 0.69-1.04) times lower than in the COVID-19–alone group (eTable 5 in Supplement 1). During a 60-day follow-up period of the immunogenicity cohort, none of its 151 participants were infected with SARS-CoV-2 (eMethods 3 in Supplement 1). A sensitivity analysis was conducted and included 2 HCWs who were excluded from the main immunogenicity analysis due having been diagnosed with a SARS-CoV-2 infection during the time between vaccine receipt and postvaccination serological testing, in which GMTs in the coadministration group were estimated to be 0.85 (95% CI, 0.69-1.05) times lower than in the COVID-19 vaccine alone group (eTable 6 in Supplement 1).

## Discussion

In this study, we assessed the reactogenicity and immunogenicity of the coadministration of COVID-19 vaccination together with SIV. Of the 3 study groups, those who received SIV alone experienced the least reactogenicity, while COVID-19 vaccination alone elicited similar reactogenicity to that of the coadministration of COVID-19 vaccine with SIV. These findings are similar to those of trials that investigated the coadministration of SIV and COVID-19 vaccines and found that coadministration of both vaccine groups was similar to COVID-19 vaccination alone in terms of adverse reactions.^4,5,7,8,9^

When assessing immunogenicity, we estimated a mild decrease in anti-spike IgG titers, with a point estimate of 16%, and a confidence interval not greater than 31%. Findings from previous immunogenicity and correlates of protection studies suggest that changes in IgG titers to such an extent did not greatly impact vaccine effectiveness,^13^ vaccine effectiveness against substantially symptomatic disease,^14^ or a SARS-CoV-2 diagnosis risk.^15^ However, the rationale behind vaccinating HCWs with booster doses against COVID-19 also takes into account the safety of the patients they are treating, and the IgG response to vaccination does not necessarily correlate with reduced infectivity of vaccinated HCWs. Current literature has conflicting reports regarding immunogenicity of COVID-19 vaccines when administered together with SIV. Some studies reported a significant decrease in postvaccination titers^9,16^ or reduced neutralization,^8^ while others found that the humoral response was similar between coadministration and COVID-19 vaccination alone.^4,5^

When looking into the current literature on the coadministration of these 2 vaccines, trials investigated the coadministration of several different types of COVID-19 vaccines. Those results may not be generalizable for the entire, very diverse, and evolving group of COVID-19 vaccines.

The preserved humoral response along with the similar reactogenic profile we found between the 2 groups lead us to conclude that the coadministration of both vaccines is an acceptable policy to increase adherence to their receipt, as adherence to a single clinic visit will surely be greater than that which can be achieved for 2 separate visits, especially in more vulnerable populations (eg, the elderly). Coadministration of vaccines is often advocated when disadvantages are marginal or negligible.^17^

### Limitations

This study has limitations. A potential limitation of our study is that the study population is composed of relatively healthy HCWs and therefore may not be fully generalizable to more vulnerable populations. Another limitation is that we only assessed the immunogenicity against SARS-CoV-2 and not the immunogenicity against influenza, which theoretically might be differently impacted by coadministration of the 2 vaccines. It should also be noted that while the analysis adjusted for it, time between vaccination and serological testing in the immunogenicity analysis varied between participants. As for the reactogenicity analysis, mean time between vaccine receipt and questionnaire response was 28.9 days, with only 9 study participants who responded after more than 45 days. This may introduce recall bias, which is another potential limitation of this study. However, we believe that even mild, but significant reactions would have been reported accurately a month after occurring. An additional limitation of the reactogenicity analysis is the 30.8% response rate to the questionnaire, which may induce a selection bias, as those who experienced more severe symptoms following vaccine receipt are potentially more likely to opt to respond to the questionnaire.

## Conclusions

In this cohort study of HCWs who were vaccinated with the Omicron BA.4/BA.5–adapted bivalent vaccine, an influenza vaccine (Influvac Tetra), or both, we found that coadministration did not lead to a substantially inferior immune response or to an increased rate of reactogenicity events compared with the administration of this COVID-19 vaccine alone. Although this may not be generalizable to other COVID-19 vaccines, and further studies on vaccine efficacy could likely shed more light on the repercussions of this practice, we believe that our results suggest that the coadministration of this COVID-19 vaccine along with SIV is a feasible and harmless tactic to increase vaccine uptake.

## Notes

Supplement 1. **eMethods 1.** Inclusion and Exclusion Criteria: Reactogenicity Analysis **eMethods 2.** Inclusion and Exclusion Criteria: Immunogenicity Analysis **eMethods 3.** Follow-Up in the Immunogenicity Cohort **eTable 1.** Reactogenicity Questionnaire **eTable 2.** Characteristics of the COVID-19 Vaccine—Alone Group Within the Immunogenicity Analysis **eTable 3.** Baseline Characteristics of Staff to Whom the Reactogenicity Questionnaire Was Sent and Who Met Eligibility Criteria **eTable 4.** Results of the Reactogenicity Analysis: Estimated Odds Ratio **eTable 5.** Adjustment Variables of the Multivariable Linear Regression Used to Assess the Geometric Mean Ratio Between the Different Study Groups: Main Analysis **eTable 6.** Adjustment Variables of the Multivariable Linear Regression Used to Assess the Geometric Mean Ratio Between the Different Study Groups—Sensitivity Analysis: Inclusion of HCW Who Tested Positive for SARS-CoV-2 After Vaccine Receipt and Before Serological Sample Collection

Supplement 2.
Data Sharing Statement
