# Supplementary material for: Immunogenicity and Reactogenicity of Coadministration of COVID-19 and Influenza Vaccines
Source: JAMA Netw Open. 2023 Sep 8;6(9):e2332813. doi: 10.1001/jamanetworkopen.2023.32813 (PMC10492184; doi:10.1001/jamanetworkopen.2023.32813)
Supplement: Supplement 1. — eMethods 1. Inclusion and Exclusion Criteria: Reactogenicity Analysis eMethods 2. Inclusion and Exclusion Criteria: Immunogenicity Analysis eMethods 3. Follow-Up in the Immunogenicity Cohort eTable 1. Reactogenicity Questionnaire eTable 2. Characteristics of the COVID-19 Vaccine—Alone Group Within the Immunogenicity Analysis eTable 3. Baseline Characteristics of Staff to Whom the Reactogenicity Questionnaire Was Sent and Who Met Eligibility Criteria eTable 4. Results of the Reactogenicity Analysis: Estimated Odds Ratio eTable 5. Adjustment Variables of the Multivariable Linear Regression Used to Assess the Geometric Mean Ratio Between the Different Study Groups: Main Analysis eTable 6. Adjustment Variables of the Multivariable Linear Regression Used to Assess the Geometric Mean Ratio Between the Different Study Groups—Sensitivity Analysis: Inclusion of HCW Who Tested Positive for SARS-CoV-2 After Vaccine Receipt and Before Serological Sample Collection [file jamanetwopen-e2332813-s001.pdf]

Supplemental Online Content

Gonen T, Barda N, Asraf K, et al. Immunogenicity and reactogenicity of coadministration of COVID-19 and influenza vaccines. *JAMA Netw Open.* 2023;6(9):e2332813. doi:10.1001/jamanetworkopen.2023.32813

eMethods 1. Inclusion and Exclusion Criteria: Reactogenicity Analysis

eMethods 2. Inclusion and Exclusion Criteria: Immunogenicity Analysis

eMethods 3. Follow-Up in the Immunogenicity Cohort

eTable 1. Reactogenicity Questionnaire

eTable 2. Characteristics of the COVID-19 Vaccine—Alone Group Within the Immunogenicity Analysis

eTable 3. Baseline Characteristics of Staff to Whom the Reactogenicity Questionnaire Was Sent and Who Met Eligibility Criteria

eTable 4. Results of the Reactogenicity Analysis: Estimated Odds Ratio

eTable 5. Adjustment Variables of the Multivariable Linear Regression Used to Assess the Geometric Mean Ratio Between the Different Study Groups: Main Analysis

eTable 6. Adjustment Variables of the Multivariable Linear Regression Used to Assess the Geometric Mean Ratio Between the Different Study Groups—Sensitivity Analysis: Inclusion of HCW Who Tested Positive for SARS-CoV-2 After Vaccine Receipt and Before Serological Sample Collection

This supplemental material has been provided by the authors to give readers additional information about their work.

© 2023 Gonen T et al. *JAMA Network Open.*

eMethods 1. Inclusion and Exclusion Criteria: Reactogenicity Analysis

- **Inclusion criteria for this analysis were:**
    a. Having received a bivalent Pfizer-BioNTech COVID-19 vaccine (BA4/BA5), and/or a SIV (Abbott Influvac Tetra) at SMC during the study's period (September 12, 2022 to December 29, 2022)
    b. Participation in the Sheba Serology cohort.
- **Exclusion criteria for this analysis were:**
    a. Immunosuppression (defined as: receiving immunosuppressive therapy, such as: biological therapies, corticosteroids, chemotherapy; having undergone a splenectomy; having been diagnosed with HIV)
    b. Having received both COVID-19 and SIV on separate days, within less than 7 days of one another.
    c. Answering the electronic questionnaire less than 4 days after having received vaccination.
    d. Partial/incoherent answers to the questionnaire (e.g., reported symptoms of COVID-19 vaccination while only having received SIV, etc).

© 2023 Gonen T et al. *JAMA Network Open.*

eMethods 2. Inclusion and Exclusion Criteria: Immunogenicity Analysis

- **Inclusion criteria for this analysis were:**
  a. having received a COVID-19 vaccine (BA4/BA5) at SMC during the study's period (September 12, 2022 to December 29, 2022), participation in the Sheba Serology cohort
  b. Having undergone serology tests before (up to 40 days) and after (6-70 days) vaccination
- **Exclusion criteria for this analysis were:**
  a. Immunosuppression (defined as: receiving immunosuppressive therapy, such as: biological therapies, corticosteroids, chemotherapy; having undergone a splenectomy; having been diagnosed with HIV)
  b. A documented diagnosis of COVID-19 during the period between COVID-19 vaccine receipt and post-vaccination serological test
  c. Having received both COVID-19 and SIV on separate days, within less than 7 days of each other.

© 2023 Gonen T et al. *JAMA Network Open.*

eMethods 3. Follow-Up in the Immunogenicity Cohort

A diagnosis of SARS-CoV-2 infection was defined as a positive PCR or rapid antigen test conducted either in the community or in SMC. Data regarding community diagnoses was provided to SMC by the Israeli Ministry of Health.

© 2023 Gonen T et al. *JAMA Network Open.*

eTable 1. Reactogenicity Questionnaire

Question	Answer 1	Answer 2	Answer 3	Answer 4	Answer 5
Which Vaccines did you recently receive at SMC?	COVID-19 Vaccine	Influenza Vaccine	Both vaccines on the same day	Both vaccines on separate days	
If you received both vaccines on separate days, please respond to the following questions regarding only the COVID-19 vaccine you had received					
Did you experience any local symptoms (such as pain, redness, swelling, or other symptoms) at the site of injection?	Yes	No			
Which arm was affected?	The one in which I received the COVID-19 vaccine	The one in which I received the influenza vaccine	Both arms	One arm only, but I cannot recall which one	
How many days did the local symptoms last?					
On a scale of 1 to 10, with 1 being mild and 10 being very severe, how would you rate the severity of your symptoms?	1-10				
Did you experience any systemic symptoms (fever, headache, myalgia, fatigue/weakness, lymphadenopathy or other)?	Yes	No			
Did you experience the following symptoms?					
Fever ≥ 37.5?	Yes	No			
If so, how many days did the fever last?					
What was the highest fever you measured?					
Significant weakness and/or fatigue?	Yes	No			
Muscle aches?	Yes	No			
Headache?	Yes	No			
How long did those symptoms (i.e, headache/muscle aches/fever ≥ 37.5/weakness or fatigue) last?	A day or less	One to two days	Two to three days	Three to four days	Four days or longer
Lymph node enlargement?	Yes	No			
Was the lymph node enlargement located to your axillary area or arm?	Yes	No			
Was the lymph node enlargement located to other body areas (jaw, neck, for example)?	Yes	No			
Generalized allergic reaction?	Yes	No			
Lab result changes (only if lab tests were performed)?	Yes	No			
Please detail which lab result changes did you have:					
Facial nerve palsy (Bell's palsy/Facialis)?	Yes	No			
Paraesthesia (tingling/numbness)?	Yes	No			
Other symptoms?	Yes	No			

© 2023 Gonen T et al. *JAMA Network Open.*

Question	Answer 1	Answer 2	Answer 3	Answer 4	Answer 5
Which other symptoms did you experience?					
Did you take a sick leave due to your symptoms?	Yes	No			
If so, for how long (days)?					
Did you seek medical attention because of your symptoms?	Yes	No			
Which symptoms led you to seek medical attention?					
Did your symptoms require a hospital admission/emergency room visit?	Yes	No			

© 2023 Gonen T et al. *JAMA Network Open.*

eTable 2. Characteristics of the COVID-19 Vaccine—Alone Group Within the Immunogenicity Analysis

**Characteristic**	**COVID-19 vaccine alone group population, N=74**
Not received SIV at all during the study's follow-up period, or received it 7 or more days after COVID-19 vaccine receipt	56 (76%)
Received SIV prior to COVID-19 vaccination	18 (24%)
Received SIV 21 or more days before receiving a COVID-19 vaccine	8 (11%)
Received SIV 14-20 days before receiving a COVID-19 vaccine	3 (4%)
Received the SIV 7 to 13 days before receiving a COVID-19 vaccine	7 (9%)

© 2023 Gonen T et al. *JAMA Network Open.*

eTable 3. Baseline Characteristics of Staff to Whom the Reactogenicity Questionnaire Was Sent and Who Met Eligibility Criteria

**Characteristic**	**Questionnaire answered (n=588)**	**Questionnaire unanswered (n=1,437)**
Average age	56.7	48.5
Sector		
Female gender	236 (40.1%)	903 (64.4%)
Physician	90 (15.4%)	424 (30.1%)
Nursing	119 (20.4%)	384 (27.3%)
Logistics and maintenance	217 (37.2%)	319 (22.7%)
Allied health professions	158 (27.1%)	280 (19.9%)
Number of vaccines previously received		
None	1 (0.2%)	4 (0.3%)
One	9 (1.5%)	55 (3.9%)
Two	20 (3.4%)	80 (5.7%)
Three	184 (31.5%)	691 (49.1%)
Four	370 (63.4%)	577 (41.0%)
Missing data	4	34

© 2023 Gonen T et al. *JAMA Network Open.*

eTable 4. Results of the Reactogenicity Analysis: Estimated Odds Ratio

Symptoms	COVID-19 vaccine alone (N=85)	SIV vaccine alone (N=357)	COVID-19 vaccine administered together with SIV (N=146)
Any local symptoms	Baseline	0.27 (95% CI: 0.15-0.47)	1.02 (95% CI: 0.57-1.82)
Any systemic symptoms	Baseline	0.17 (95% CI: 0.09-0.33)	0.82 (95% CI: 0.43-1.56)
Fever ≥ 37.5	Baseline	0.15 (95% CI: 0.04-0.64)	1.13 (95% CI: 0.37-3.65)
Significant weakness or fatigue	Baseline	0.22 (95% CI: 0.11-0.48)	1.15 (95% CI: 0.57-2.32)
Duration of headache, myalgia, significant weakness, or fatigue	Baseline	0.17 (0.08-0.35)	0.83 (0.42-1.62)

© 2023 Gonen T et al. *JAMA Network Open.*

eTable 5. Adjustment Variables of the Multivariable Linear Regression Used to Assess the Geometric Mean Ratio Between the Different Study Groups: Main Analysis

**Variable**	
Time from vaccination to IgG test	
14-20 days	1.41 (95% CI: 1.09-1.83)
21-27 days	1.43 (95% CI: 1.12-1.82)
28-34 days	1.3 (95% CI: 1.01-1.67)
35-41 days	1.11 (95% CI: 0.83-1.49)
42-60 days	1.17 (95% CI: 0.93-1.47)
Age	1 (95% CI: 1-1.01)
Male gender	1.02 (95% CI: 0.82-1.27)
Log of pre-vaccination IgG	2.65 (95% CI: 2.25-3.13)

© 2023 Gonen T et al. *JAMA Network Open.*

eTable 6. Adjustment Variables of the Multivariable Linear Regression Used to Assess the Geometric Mean Ratio Between the Different Study Groups—Sensitivity Analysis: Inclusion of HCW Who Tested Positive for SARS-CoV-2 After Vaccine Receipt and Before Serological Sample Collection

Variable	
Time from vaccination to IgG test	
14-20 days	1.41 (95% CI: 1.09-1.82)
21-27 days	1.43 (95% CI: 1.13-1.83)
28-34 days	1.29 (95% CI: 1.01-1.66)
35-41 days	1.11 (95% CI: 0.83-1.49)
42-60 days	1.17 (95% CI: 0.93-1.47)
Age	1.01 (95% CI: 1-1.01)
Male gender	1.00 (95% CI: 0.81-1.24)
Log of pre-vaccination IgG	2.64 (95% CI: 2.24-3.12)
Receipt of both vaccines on the same day	0.85 (95% CI: 0.69-1.05)

© 2023 Gonen T et al. *JAMA Network Open.*
