# Supplementary material for: Immunogenicity and Reactogenicity of Coadministration of COVID-19 and Influenza Vaccines
Source: JAMA Netw Open. 2023 Sep 8;6(9):e2332813. doi: 10.1001/jamanetworkopen.2023.32813 (PMC10492184; doi:10.1001/jamanetworkopen.2023.32813)
Supplement: Supplement 2. — Data Sharing Statement [file jamanetwopen-e2332813-s002.pdf]

Data Sharing Statement

Data
Data available: Yes
Data types: Deidentified participant data
How to access data: Gili.Regev@sheba.health.gov.il
When available: With publication

Supporting Documents
Document types: None

Additional Information
Who can access the data: Researchers whose proposed use of the data has been approved
Types of analyses: Public health research
Mechanisms of data availability: With investigator support
